# Epigenomic states contribute to coordinated allelic transcriptional bursting in iPSC reprogramming

**DOI:** 10.26508/lsa.202302337

**Published:** 2024-02-06

**Authors:** Parichitran Ayyamperumal, Hemant Chandru Naik, Amlan Jyoti Naskar, Lakshmi Sowjanya Bammidi, Srimonta Gayen

**Affiliations:** Chromatin, RNA and Genome (CRG) Laboratory, Department of Developmental Biology and Genetics, Indian Institute of Science , Bangalore, India

## Abstract

Two alleles of a gene can be transcribed independently or coordinatedly, which can lead to temporal expression heterogeneity with potentially distinct impacts on cell fate. Here, we profiled genome-wide allelic transcriptional burst kinetics during the reprogramming of MEF to induced pluripotent stem cells. We show that the degree of coordination of allelic bursting differs among genes, and alleles of many reprogramming-related genes burst in a highly coordinated fashion. Notably, we show that the chromatin accessibility of the two alleles of highly coordinated genes is similar, unlike the semi-coordinated or independent genes, suggesting the degree of coordination of allelic bursting is linked to allelic chromatin accessibility. Consistently, we show that many transcription factors have differential binding affinity between alleles of semi-coordinated or independent genes. We show that highly coordinated genes are enriched with chromatin accessibility regulators such as H3K4me3, H3K4me1, H3K36me3, H3K27ac, histone variant H3.3, and BRD4. Finally, we demonstrate that enhancer elements are highly enriched in highly coordinated genes. Our study demonstrates that epigenomic states contribute to coordinated allelic bursting to fine-tune gene expression during induced pluripotent stem cell reprogramming.

## Abstract (precis)

Epigenomic states are linked to the coordinated allelic transcriptional bursting of genes involved in iPSC reprogramming.

## Introduction

Reprogramming of somatic cells to induced pluripotent stem cells (iPSCs) serves as an excellent model system for studying the mechanisms of cell fate specification and gene regulation (Takahashi & Yamanaka, 2006, 2016; Onder et al, 2012; Polo et al, 2012; Cheloufi et al, 2015; Bauer et al, 2021; Generoso et al, 2023; Naik et al, 2023 *Preprint*). However, mechanisms of iPSC reprogramming remain poorly understood. Precise expression of genes involved in iPSC reprogramming is crucial for reprogramming. To date, the gene expression dynamics during iPSC reprogramming have been studied at the gene level but not at the allelic level. In eukaryotic cells, transcription happens in a sporadic manner through random transcriptional bursting separated by periods of silent state, which contributes to the gene expression heterogeneity among the identical cells (Raj et al, 2006; Raj & van Oudenaarden, 2008; Little et al, 2013; Padovan-Merhar et al, 2015; Larsson et al, 2019b; Naik et al, 2021; RV et al, 2021). On the other hand, the regulation of transcriptional bursting can be shared or autonomous between alleles (Onuchic et al, 2018; Finn et al, 2019; Naik et al, 2021; RV et al, 2021). Indeed, recent studies by us and others demonstrated that often the kinetics of transcriptional bursting of the two alleles of a gene differs, which in turn gives rise to dynamic autosomal random monoallelic expression (aRME) (Borel et al, 2015; Reinius & Sandberg, 2015; Gendrel et al, 2016; Reinius et al, 2016; Gregg, 2017; Naik et al, 2021; RV et al, 2021). It is believed that dynamic aRME can induce temporal variations of gene expression among cells and thereby may contribute to cell fate specification. Therefore, it is important to profile the transcriptional kinetics of genes at the allelic level to understand how gene expression is fine-tuned for precise cell fate specification. However, allelic transcriptional kinetics during iPSC reprogramming remains unexplored. To address this, we have profiled genome-wide transcriptional burst kinetics at allelic level across different stages of reprogramming of MEF to iPSC using allele-specific single-cell RNA-sequencing (scRNA-seq) analysis. We find that the degree of coordination of allelic bursting differs among genes and changes dynamically during iPSC reprogramming. Importantly, we find that many genes involved in reprogramming pathways have a high degree of allelic coordination. On the other hand, the factors involved in coordinating allelic bursting are not known. Transcription factors and enhancer functions are thought to modulate transcriptional burst kinetics (Larsson et al, 2019b). Emerging trends suggest that epigenomic states are linked to burst kinetics (Nicolas et al, 2018; Chen et al, 2019; Fraser et al, 2021). Therefore, we have explored how epigenomic features contribute to the coordinated allelic transcriptional bursting during iPSC reprogramming.

## Result

### Prevalent bursty expression contributes to dynamic aRME during iPSC reprogramming

**Figure 1.**
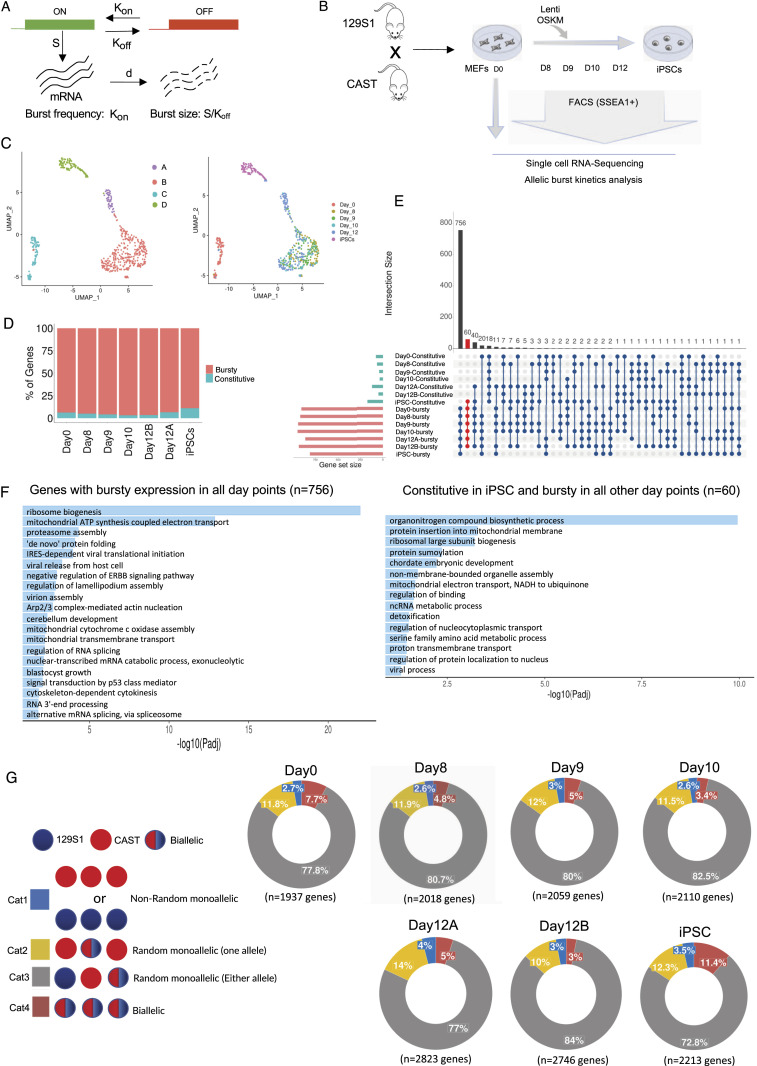
Genome-wide profiling of bursty expression and dynamic autosomal random monoallelic expression in different stages of MEF to induced pluripotent stem cell (iPSC) reprogramming. **(A)** A diagrammatical representation of the “two-state model” of transcriptional bursting. K_on_ is the rate of transcriptional activation of a gene; K_off_ is the rate at which a gene becomes transcriptionally inactive; S is the rate of transcription of a gene in an active state; d is the rate of mRNA decay; burst kinetics is represented through K_on_ (burst frequency) and S/K_off_ (burst size). **(B)** Graphical representation of OSKM (Oct-3/4, Sox2, Klf4, and c-Myc)-mediated reprogramming of hybrid MEF cells (129S1 X CAST) and collection of cells of intermediate stages day 8, day 9, day 10, day 12, and iPSCs. **(C)** Plots representing UMAP-based clustering of cells of the different stages of reprogramming. **(D)** Quantification of the percent of genes exhibiting bursty expression across all-day points of reprogramming. The number of bursty genes in day 0: 1,769 (of 1,886 biallelic genes), day 8: 1,869 (of 1,968 biallelic genes), day 9: 1,921 (of 2,004 biallelic genes), day 10: 1,993 (of 2,058 biallelic genes), day 12A: 2,544 (of 2,723 biallelic genes), day 12B: 2,585 (of 2,678 biallelic genes), and iPSC: 1,901 (of 2,137 biallelic genes). **(E)** Cross-comparison plot of bursty and constitutive genes across the different stages of reprogramming. **(F)** Gene ontology (GO) enrichment analysis of 756 genes that remain bursty across all-day points and 60 genes that are constitutive in iPSCs but bursty in other-day points. **(G)** Plots representing the percent of genes with different allelic expression categories throughout different stages of reprogramming; Cat 1: nonrandom monoallelic, Cat 2: random monoallelic with one allele, Cat 3: random monoallelic with either allele, Cat 4: biallelic.

**Figure S1.**
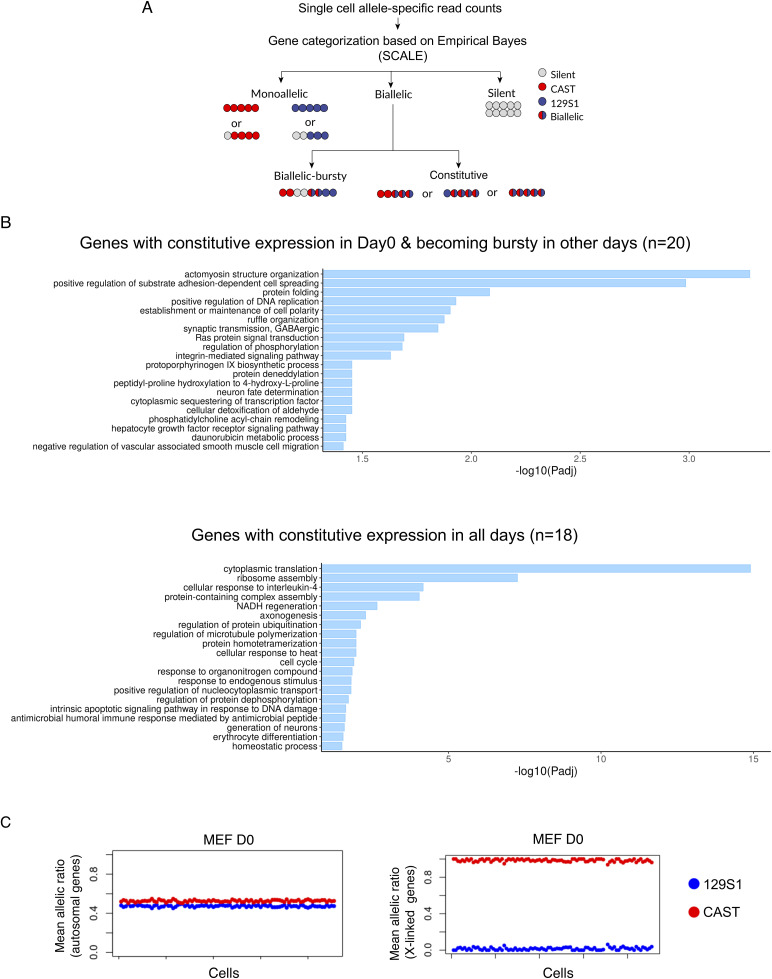
Related to Fig 1. **(A)** Outline of categorization of bursty and constitutive genes through SCALE analysis. **(B)** Gene ontology analysis. **(C)** Plots representing allele-specific expression of autosomal and X-linked genes in MEF(d0) cells.

Transcription of many genes occurs in a stochastic manner, where genes undergo sporadic bursting to produce RNA. The kinetics of transcriptional bursting are deduced through the well-known “two-state model” of transcription. According to the “two-state model,” the promoter of a gene switches stochastically from an inactive/OFF state to an active/ON state, and burst kinetics is determined through two parameters: burst frequency and burst size (Fig 1A) (Chubb et al, 2006; Raj & van Oudenaarden, 2008; Larson, 2011). Burst frequency is defined by the number of bursts per unit time, and burst size explains the average amount of mRNA molecules per burst (Fig 1A). To date, burst kinetics have been studied mainly at the gene level, not the allelic level. However, profiling burst kinetics at allelic resolution is important as the kinetics of bursting between alleles often differ (Naik et al, 2021). Therefore, to understand how individual alleles of a gene contribute to the overall gene expression, we have delineated the kinetics of allelic bursting genome-wide across different stages of reprogramming of MEF to iPSC (Fig 1B). To profile, genome-wide allelic transcriptional burst kinetics, we performed SCALE (Single-Cell ALlelic Expression) analysis using scRNA-seq datasets (Fig 1B). SCALE relies on the Empirical Bayes Framework, which first classifies the genes into monoallelic, biallelic, and silent based on the allele-specific read counts across the cells. Subsequently, the biallelic genes are further categorized into biallelic bursty and biallelic constitutive (Fig S1A) (Jiang et al, 2017). Biallelic genes that have zero expression of both alleles in a few cells but not all cells are considered bursty genes, whereas genes that have at least one allele constitutive expression or both allele constitutive expression across cells are considered biallelic constitutive genes (Fig S1A). The MEF cells used in the experiment were derived from a cross of two divergent mouse strains *M. Musculus* (129S1) and *M. Castaneous* (CAST), thereby enabling us to perform allele-specific analysis based on strain-specific SNPs (Fig 1B) (Talon et al, 2021). We excluded low-expressed genes from our study to avoid allelic dropout-related technical noise, which could lead to inaccurate estimation of the allelic expression (Kim et al, 2015; Santoni et al, 2017; Zhao et al, 2017).

First, we performed UMAP-based clustering to identify homogeneous cell populations across the different stages of reprogramming. We found that day 0-MEF cells and iPSC cells formed individual tight clusters (Fig 1C). Intermediate cells (day 8, day 9, day 10) also clustered together. However, day 12 cells formed two different sub-clusters (day 12A and day 12B). Therefore, we performed SCALE analysis separately for day 12A and day 12B sub-clusters to reduce the effect of heterogeneity in our burst kinetics analysis (Fig 1C). Through SCALE analysis, we found that most of the biallelic genes (∼90%) have bursty expression across all stages of reprogramming (Fig 1D). Next, we compared the bursty/constitutive pattern of gene expression across different reprogramming stages and found that most genes (n = 756) maintained bursty expression throughout the reprogramming (Fig 1E). Interestingly, these genes were enriched toward key reprogramming-related processes such as ribosome biogenesis, proteasome assembly, blastocyst growth, etc. (Fig 1F). Moreover, genes (n = 60) that remained bursty on all days except iPSCs were also enriched in many reprogramming-related biological processes, including chordate embryonic development (Fig 1F). These results indicated that many genes involved in iPSC reprogramming pathways exhibit bursty expression. However, a few genes (n = 20) that maintained bursty expression in all-day points except MEF did not show enrichment towards major iPSC reprogramming pathways (Fig S1B). Genes (n = 18), which remained constitutive throughout the reprogramming, were not enriched to major reprogramming pathways (Fig S1B). On the other hand, we have previously shown that bursty expression results in dynamic aRME, which creates gene expression heterogeneity among cells (Naik et al, 2021). Therefore, we explored the landscape of allelic expression throughout the different stages of iPSC reprogramming by allele-specific scRNA-seq analysis. We categorized a gene as monoallelic within a cell if at least 95% of the expression originated from one allele. We classified genes based on their allelic expression patterns into four categories: Cat 1: nonrandom monoallelic (same allele expressed across the cells); Cat 2: RME with one allele (genes expressed biallelically in some cells whereas exhibiting monoallelic expression from one allele consistently in other cells); Cat 3: random monoallelic with either allele (genes expressed biallelically in some cells whereas exhibiting monoallelic expression from either of the allele in other cells); and Cat 4: biallelic (genes expressed biallelically across the cells) (Fig 1G). Our analysis revealed that ∼80–90% of genes have dynamic aRME across different stages of reprogramming (Fig 1G). Analysis of allelic expression of X-linked genes in MEF showed monoallelic expression from the CAST allele as 129S1-X is inactivated in these cells, thereby validating our allele-specific analysis pipeline (Fig S1C). X-inactivation is a process through which female mammals compensate for the dosage of X-linked gene expression between sexes (Gayen et al, 2015, 2016; Sarkar et al, 2015; Saiba et al, 2018; Kaur et al, 2020; Samanta et al, 2022). Altogether, our analysis suggested widespread bursty expression of genes during iPSC reprogramming, resulting in dynamic aRME.

### Alleles of genes exhibit similar burst kinetics but have different coordination during iPSC reprogramming

**Figure 2.**
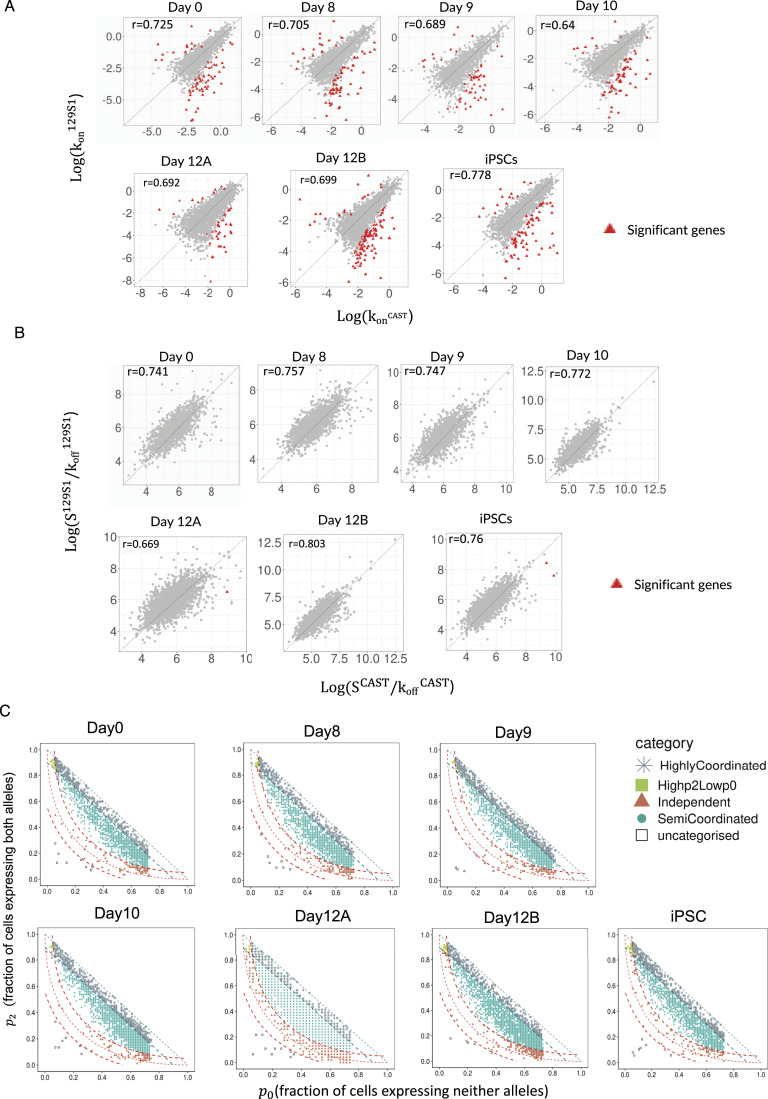
Profiling of allelic burst kinetics and coordination. **(A, B)** Plots representing correlation between (A) allelic burst frequency across different stages of reprogramming day 0 MEF (r = 0.725), day 8 (r = 0.705), day 9 (r = 0.689), day 10 (r = 0.64), day 12A (r = 0.69), day 12B (r = 0.69) and induced pluripotent stem cells (iPSCs) (r = 0.778) and (B) allelic burst size in day 0 MEF (r = 0.741), day 8 (r = 0.757), day 9 (r = 0.747), day 10 (r = 0.772), day 12A (r = 0.66), day 12B (r = 0.80) and iPSCs (r = 0.76). Genes that exhibit significant differences in burst frequency and size between two alleles have been marked by red triangles. **(C)** Smooth scatterplots representing bursting coordination between two alleles of genes for day 0 MEF, day 8, day 9, day 10, day 12A, day 12B, and iPSCs. Percent of cells expressing neither allele (p0) is plotted with the percent of cells expressing both alleles (p2); the blue diagonal line represents perfect coordination (p0 + p2 = 1), whereas the red curve signifies independent bursting with shared kinetics. Different categories of genes based on allelic bursting coordination: low p0 high p2 (green filled squares), perfectly coordinated (p0 + p2 > 0.90 marked by gray asterisk between blue dotted diagonal lines), independent genes marked by rosewood triangles (between upper and lower red curved lines, with a threshold of +0.05 signified by upper red curve and −0.05, signified by the lower red curve), and semi-coordinated genes marked with persian blue dots.

Next, we investigated if the two alleles of a gene have similar burst kinetics or not. To explore this, we profiled burst frequency and burst size at the allelic level for the biallelic bursty genes using SCALE. SCALE relies on a “two-state model” of transcription where a gene switches from active to inactive state with activation rate of Kon and deactivation rate of Koff. When a gene is in the active state, the rate of transcription is S and the rate of RNA decay is d (Fig 1A). Burst frequency is determined by the number of bursts per unit time (Kon), and burst size is deduced through average number of mRNA molecules per burst when the gene is in active state (S/Koff) (Fig 1A). We observed a high degree of correlation of both burst frequency (r = 0.64–0.778) and burst size (r = 0.669–0.803) between two alleles across all-day points (Fig 2A and B). Very few genes exhibited significant burst frequency and size differences between the two alleles, as marked by the red triangles (Fig 2A and B). Taken together, our results suggest that alleles of most of the genes have similar burst kinetics. Next, we explored the degree of coordination of bursting between two alleles by plotting the percent of cells expressing neither allele (p_0_) versus the percent of cells expressing both alleles (p_2_) (Fig 2C). Blue diagonal line represents perfect coordination (p0 + p2 = 1), whereas the red curve signifies independent bursting with shared kinetics (Fig 2C). We categorized genes based on the degree of coordination of allelic bursting into three categories: (1) highly coordinated genes: with p0 + p2 > 0.90 marked by gray asterisk between blue dotted diagonal lines; (2) independent genes: genes near the red curve with a threshold of +0.05, signified by the upper red curve, and −0.05, signified by the lower red curve, marked by rosewood triangles; (3) semi-coordinated genes that lie between the uppermost red curve line and lower blue dotted diagonal line (Persian blue dots) (Fig 2C).

We observed that most of the genes exhibited semi-coordinated allelic bursting at all-day points. However, many genes also showed highly coordinated allelic bursting across the different stages of reprogramming (Fig 2C).

### Genes involved in iPSC reprogramming undergo highly coordinated allelic bursting

**Figure 3.**
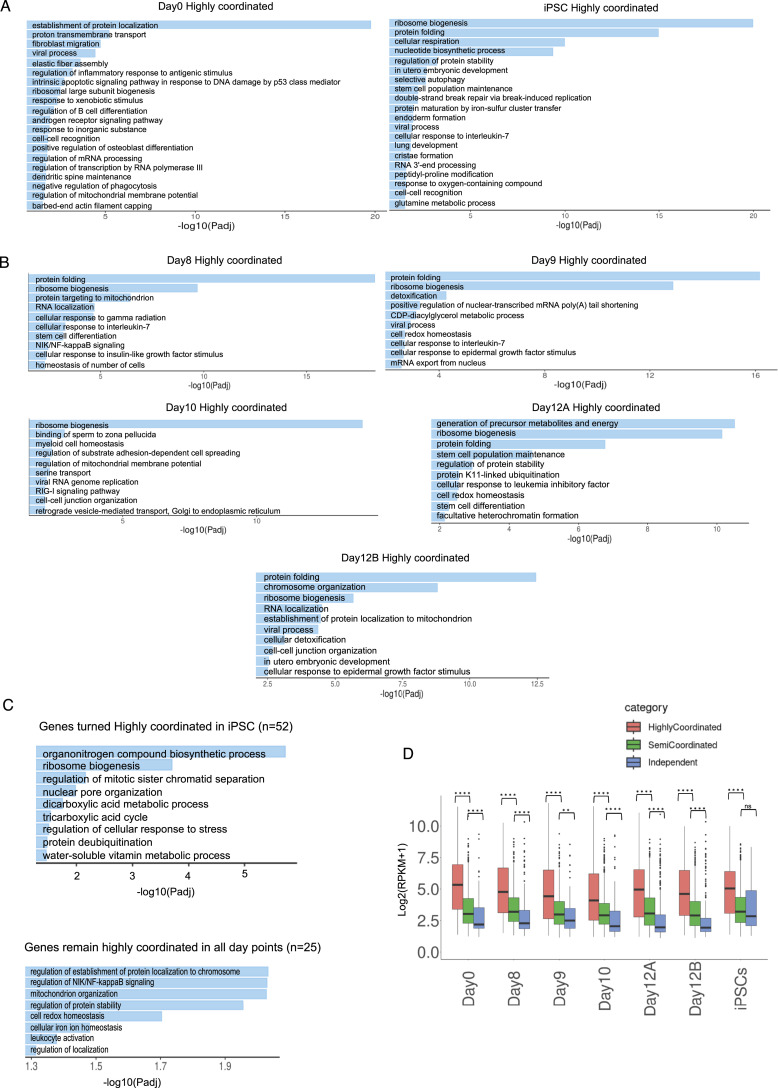
Highly coordinated genes are enriched to induced pluripotent stem cell (iPSC) reprogramming-related processes. **(A)** Gene ontology (GO) enrichment analysis of highly coordinated genes in day 0 MEF and iPSCs. **(B)** Gene ontology (GO) enrichment analysis of highly coordinated genes in intermediate stages of reprogramming: day 8, day 9, day 10, and day 12 cells. **(C)** Gene ontology (GO) enrichment analysis of genes (n = 52) that become highly coordinated in iPSCs (top) and genes (n = 25) that maintain highly coordinated allelic bursting through all-day points. **(D)** Plot representing the expression level (reads per kilobase million) of highly coordinated, semi-coordinated, and independent genes across different stages of reprogramming (Mann–Whitney *U* test: *P*-value < 0.0001; **** *P*-value < 0.01; **).

**Figure S2.**
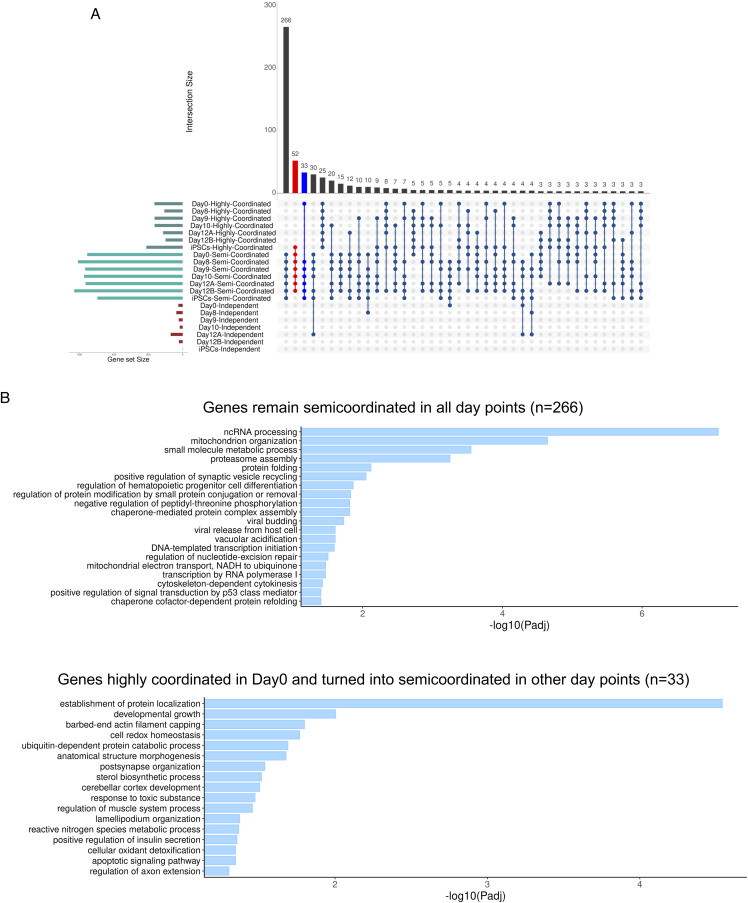
Related to Fig 3. **(A)** Cross-comparison plot of highly coordinated, semi-coordinated and independent genes across day 0 MEF, day 8, day 10, day 12, and induced pluripotent stem cells. **(B)** Gene ontology (GO) enrichment analysis of genes (n = 266) that remained semi-coordinated in all-day points (top) and highly coordinated genes (n = 33) in day 0 MEF that became semi-coordinated in other stages.

Next, we performed a gene ontology (GO) enrichment analysis of highly coordinated genes across all stages of reprogramming (Fig 3). Interestingly, we found that highly coordinated genes in iPSCs are enriched in processes linked to stem cell population maintenance, in-utero embryo development, endoderm formation, etc. (Fig 3A, Supplemental Data 1). Furthermore, highly coordinated genes in iPSCs are enriched in cellular respiration, cristae formation, and glutamine metabolism, which are relevant to metabolic remodeling in iPSCs (Fig 3A) (Teslaa & Teitell, 2015; Mathieu & Ruohola-Baker, 2017; Tohyama et al, 2017). Importantly, genes involved in ribosome biogenesis, crucial for stem cell maintenance, were highly enriched into the highly coordinated gene cohort in iPSC (Fig 3A) (Gabut et al, 2020). On the contrary, highly coordinated genes in day 0 MEF cells were not enriched in such related pathways. Interestingly, across the different intermediate stages of reprogramming, highly coordinated genes were enriched towards many reprogramming-related biological processes such as ribosome biogenesis, protein folding, stem cell maintenance, NFkB signaling, etc. (Fig 3B) (Gabut et al, 2020; Yan et al, 2020; Kaltschmidt et al, 2021). Next, we performed a cross-comparison of the three allelic coordination categories across all-day points and found that, whereas many genes maintained a similar degree of allelic coordination across all-day points, some genes did not (Fig S2A). Strikingly, we found that the genes (n = 52) that become highly coordinated in iPSCs are enriched towards ribosome biogenesis, aerobic respiration, nuclear pore formation, which are highly critical and directly relevant for iPSC reprogramming (Fig 3C) (Xu et al, 2013; Teslaa & Teitell, 2015). Moreover, genes that remain highly coordinated (n = 25) on all days showed enrichment towards some iPSC reprogramming-linked functions like protein stability, NFkB pathway, mitochondrion organization, etc. (Fig 3C). Altogether, our analysis revealed that the two alleles of many genes involved in iPSC reprogramming have a high degree of transcriptional bursting coordination (Supplemental Data 1). In parallel, we also observed that genes that remained semi-coordinated (n = 266) on all days or converted from highly coordinated on day 0 to semi-coordinated on other days (n = 33) were enriched towards some iPSC reprogramming-related pathways like proteasome assembly, oxidative stress response, etc. (Fig S2B) (Szutorisz et al, 2006; Schröter & Adjaye, 2014; Hawkins et al, 2016; Mathieu & Ruohola-Baker, 2017). Next, we investigated if higher coordination of transcriptional bursting between alleles of individual genes facilitates the optimal expression dosage of these highly coordinated genes involved in iPSC reprogramming. To explore this, we compared the overall expression level of highly coordinated, semi-coordinated, and independent genes. We found that the indeed highly coordinated genes have higher expression level compared to the semi-coordinated and independent genes (Fig 3D). Taken together, we conclude that highly coordinated allelic bursting helps to fine-tune the optimal expression of genes involved in iPSC reprogramming.

Supplemental Data 1. GO analysis of the different categories of genes during induced pluripotent stem cell reprogramming.

### Coordinated allelic bursting is linked to chromatin accessibility

**Figure 4.**
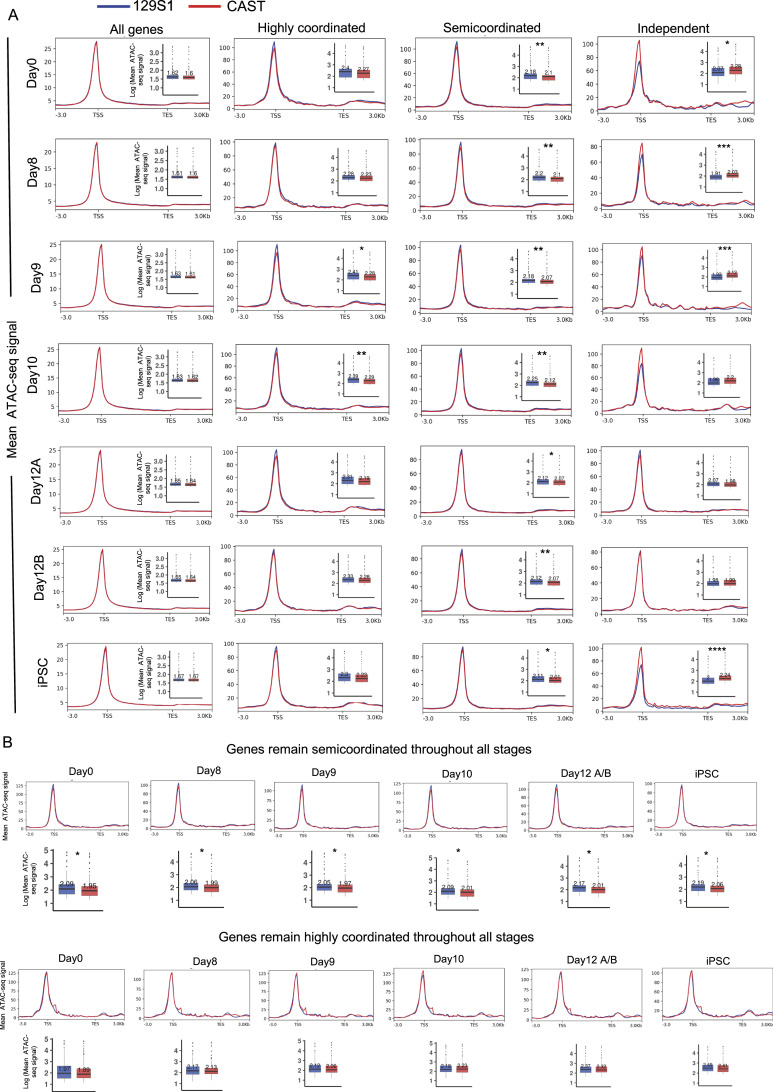
Comparison of allelic chromatin accessibility of highly coordinated, semi-coordinated, and independent genes. **(A)** Quantitative analysis of allelic accessibility enrichment in the gene body and across 3 kb upstream of TSS and 3 kb downstream of TES of all autosomal genes, highly coordinated, semi-coordinated, and independent genes throughout different stages of reprogramming: day 0, day 8, day 10, day 12, and induced pluripotent stem cells. In the boxplots, the line inside each of the boxes denotes the median value, and the edges of each box represent 25% and 75% of dataset, respectively (Mann–Whitney *U* test: *P*-value < 0.0001; **** *P*-value < 0.001; *** *P*-value < 0.01; ** and *P*-value < 0.05; *). **(B)** Allelic accessibility enrichment analysis in the gene body and across 3 kb upstream of TSS and 3 kb downstream of TES of genes that remain semicordinated or highly coordinated throughout the reprogramming (Mann–Whitney *U* test: *P*-value < 0.05; *).

**Figure S3.**
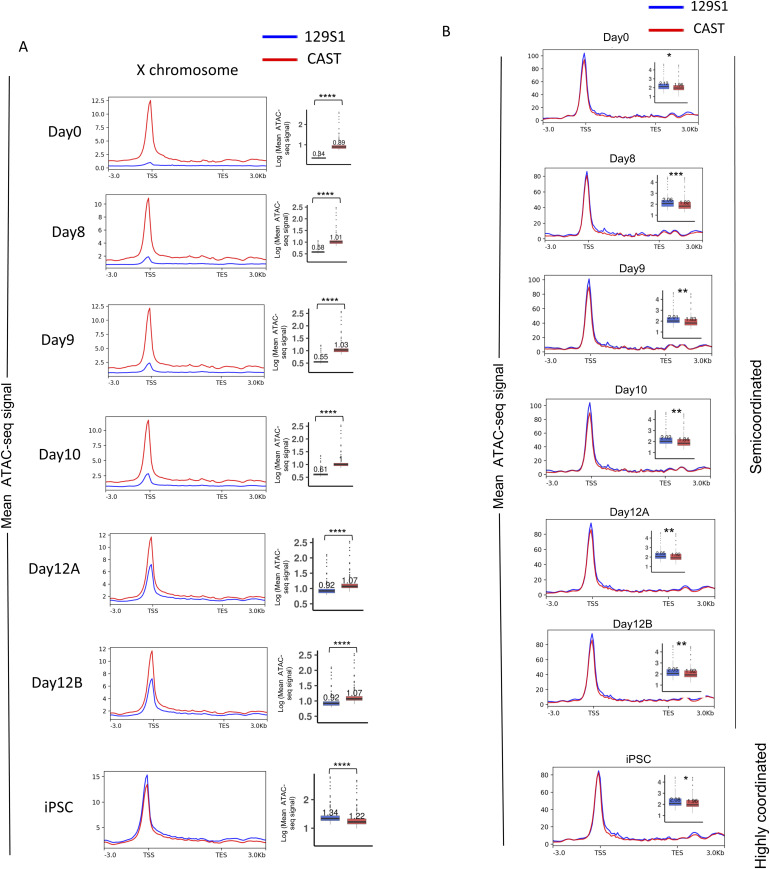
Related to Fig 4. **(A)** Allelic chromatin accessibility for X-linked genes. Quantification of enrichment of allelic accessibility across the gene body and 3 kb upstream of TSS and 3 kb downstream of TES of X-linked genes in all-day points of reprogramming. In the boxplots, the line inside of each box signifies median value, whereas the edges of each box denote 25% and 75% of the datasets (Mann-Whitney *U* test: *P*-value < 0.0001; ****). **(B)** Plots representing allelic accessibility enrichment for genes that converted from semi-coordinated in all days to highly coordinated in induced pluripotent stem cells. In the boxplots, the line inside each of the boxes denotes the median value, and the edges of each box represent 25% and 75% of the dataset, respectively (Mann–Whitney *U* test: *P*-value < 0.001; *** *P*-value < 0.01; ** and *P*-value < 0.05; *).

To understand the mechanisms of allelic bursting coordination, we asked whether the degree of coordination of allelic bursting is linked to epigenomic states. To address this, we profiled genome-wide allelic chromatin accessibility across different stages of MEF to iPSC reprogramming through allele-specific analysis of available ATAC-sequencing (ATAC-seq) datasets (Talon et al, 2021). The same hybrid MEFs (129S1X CAST), as described for scRNA-seq, were used for this experiment, allowing us to profile chromatin accessibility at the allelic level. We analyzed ATAC-seq in MEFs (day 0) and across reprogramming stages (SSEA1+ reprogramming intermediates at days 8, 9, 10, 12, and iPSCs), like the burst kinetics analysis (Fig 4A). We first validated our allele-specific ATAC-seq analysis pipeline by quantifying the difference in the enrichment of ATAC-seq reads between active-X (129S1) versus inactive-X (CAST allele) (Fig S3A). In consistence with previous reports, in MEFs and early reprogramming intermediates, active-X (CAST) showed strong enrichment of ATAC-seq reads, whereas the inactive-X (129S1) showed almost no enrichment, and upon reactivation of the inactive-X towards the attainment of iPSCs, there was a gain of chromatin accessibility (Fig S3A). Taken together, enrichment analysis of ATAC-seq reads of X-linked genes validated the accuracy of our method. Next, we compared the enrichment of ATAC-seq reads between two alleles of different categories of genes (highly coordinated, semi-coordinated, and independent) across the gene body and 3 kb upstream of TSS and 3 kb downstream of TES during reprogramming (Fig 4A). Interestingly, our analysis revealed that the two alleles of highly coordinated genes have very similar enrichment at most day points. Whereas enrichment of ATAC-seq reads of alleles of semi-coordinated/independent genes differed in most cases (Fig 4A). As expected, allelic enrichment of ATAC-seq reads considering all autosomal genes was quite similar (Fig 4A).

Notably, genes that maintained semi-coordinated bursting throughout reprogramming maintained differences in allelic accessibility (Fig 4B). Similarly, genes that maintained highly coordinated bursting throughout reprogramming always maintained similar allelic accessibility (Fig 4B). Furthermore, we show that allelic accessibility differences reduce upon becoming highly coordinated in iPSC after maintaining a semi-coordinated bursting throughout the other day points (Fig S3B). A similar trend was found for genes which switched from highly coordinated in MEF to semi-coordinated on other days (data not shown). Together, our analysis suggested a positive correlation between the coordination of allelic bursting and the similarity of allelic chromatin accessibility.

**Figure 5.**
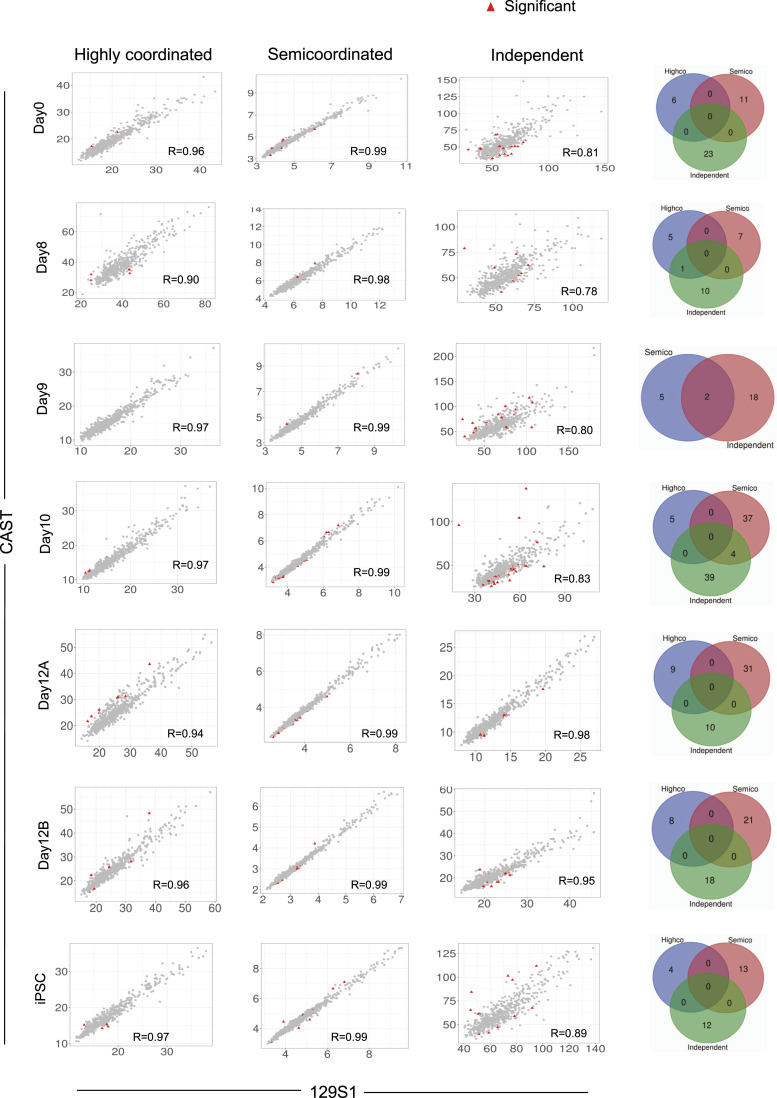
Binding kinetics of TFs between alleles correlate with the allelic bursting coordination. Left: Plots showing the correlation of TFs binding score between alleles of highly coordinated, semi-coordinated, and independent genes throughout different stages of reprogramming: day 0, day 8, day 10, day 12, and induced pluripotent stem cells. TFs that exhibit significant differences in binding between two alleles have been marked by red triangles. Right: Venn diagram representing the comparison of TFs that exhibit significant differences in binding between two alleles across highly coordinated, semi-coordinated, and independent genes.

Next, we explored if allelic accessibility differences in semi-coordinated and independent genes are associated with the differential binding of transcription factors (TFs) between alleles of individual genes. To test this, we determined TFs binding scores of individual alleles of a gene using TOBIAS (Transcription factor Occupancy prediction By Investigation of ATAC-seq Signal). Interestingly, we found that many TFs had a significantly different binding score between individual alleles of semi-coordinated and independent genes, which was not the case for highly coordinated genes (Fig 5). Taken together, our analysis suggests that allelic accessibility differences in semi-coordinated or independent genes allow differential binding of certain TFs among alleles of genes, which in turn leads to semi-coordinated or independent transcriptional bursting.

### Chromatin accessibility factors contribute to allelic bursting coordination

**Figure 6.**
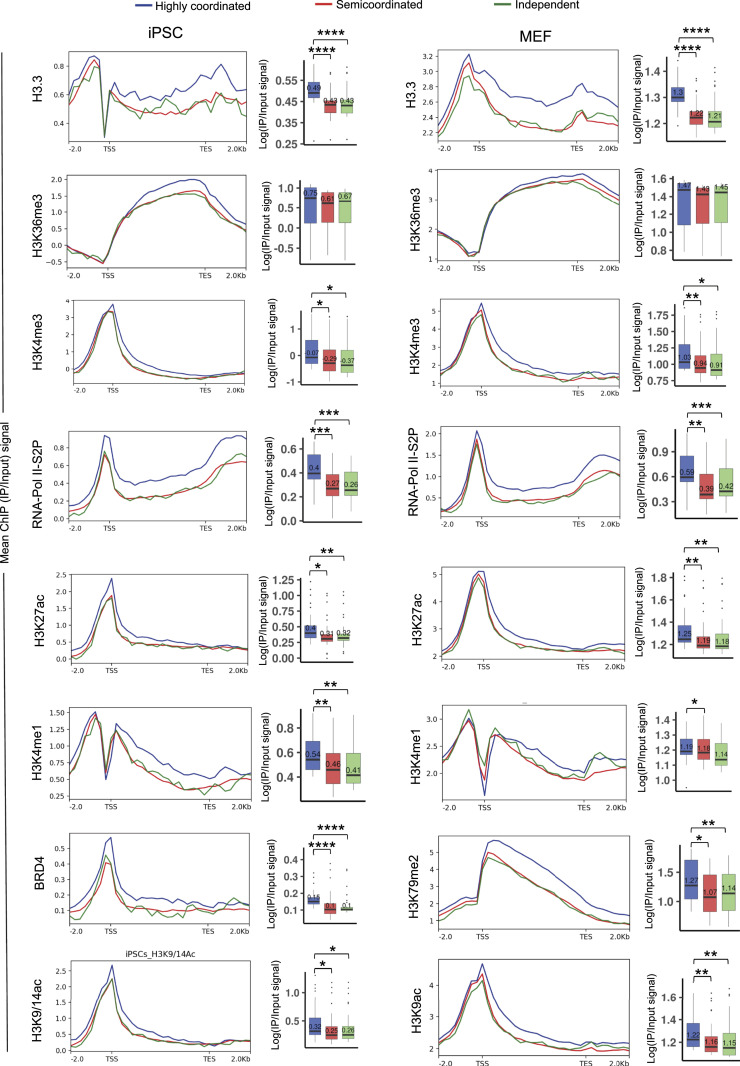
Correlation between occupancy of chromatin marks and degree of allelic bursting coordination. Comparison of enrichment of different chromatin accessibility-related factors in the gene body and across 2 kb upstream of TSS and 2 kb downstream of TES of highly coordinated, semi-coordinated, and independent genes in MEF and induced pluripotent stem cells. In the boxplots, the line inside each of the boxes denotes the median value and the edges of each box represent 25% and 75% of dataset, respectively (Mann–Whitney *U* test: *P*-value < 0.0001; **** *P*-value < 0.001; *** *P*-value < 0.01; ** and *P*-value < 0.05; *).

**Figure S4.**
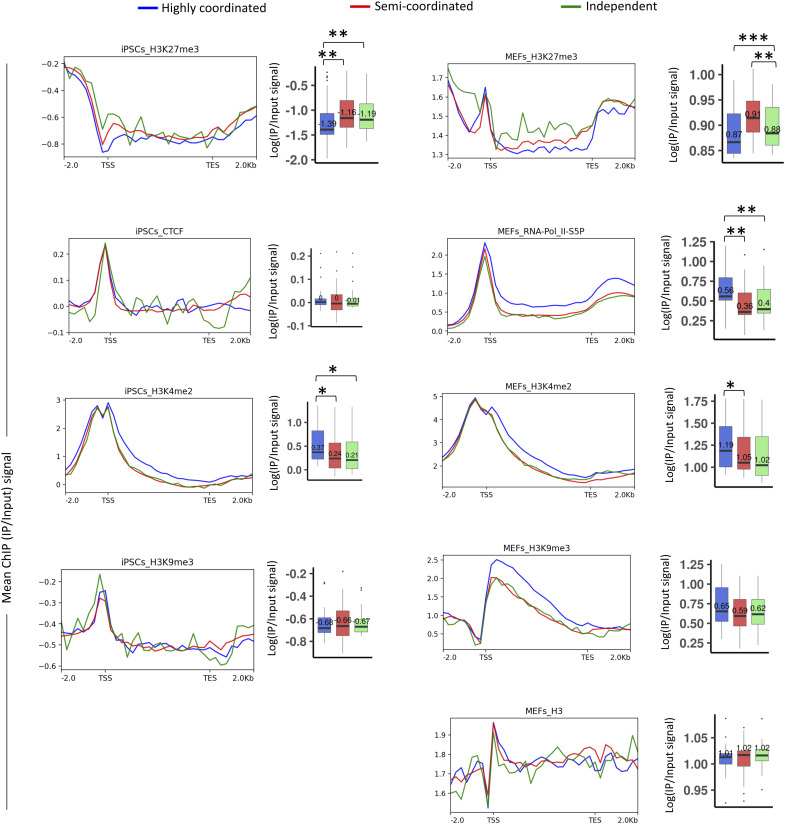
Related to Fig 6. Comparison of enrichment of different chromatin accessibility related factors in the gene body and across 2 kb upstream of TSS and 2 kb downstream of TES of highly coordinated, semi-coordinated, and independent genes in MEF and induced pluripotent stem cells.

**Figure S5.**
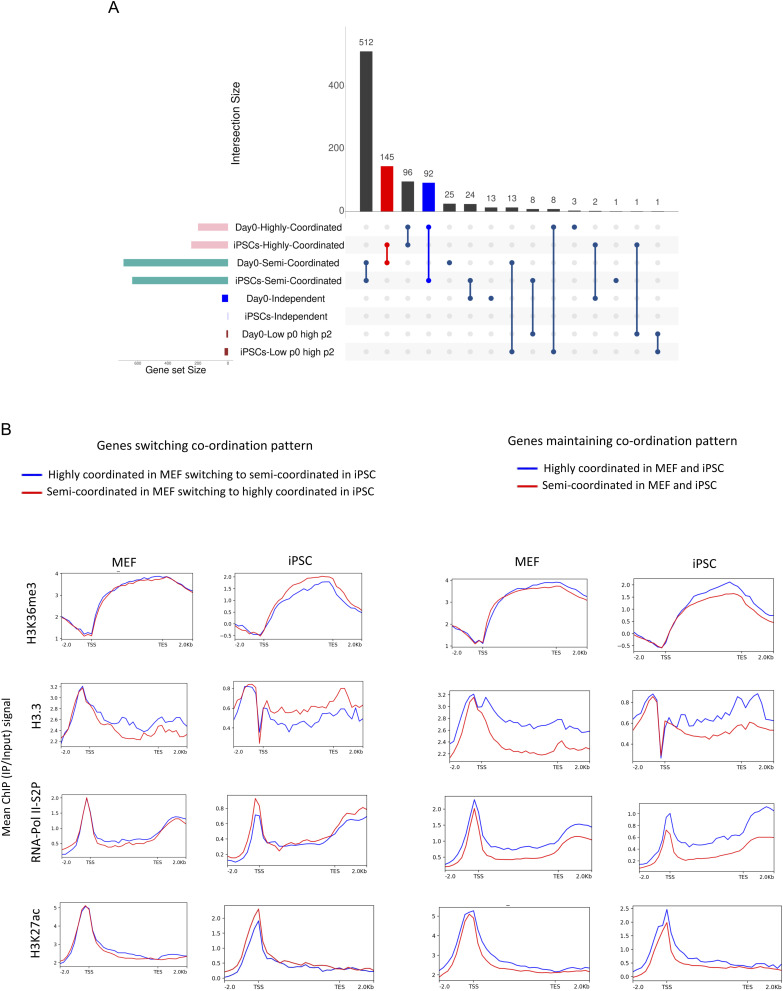
Related to Fig 6. **(A)** Cross-comparison plot of highly coordinated, semi-coordinated, and independent genes across day 0 MEF and induced pluripotent stem cells. **(B)** Plots representing quantitative enrichment of different chromatin accessibility-related factors (H3K27ac, H3.3, H3K36me3, and RNA PolII-S2P) in the gene body and across 2 kb upstream of TSS and 2 kb downstream of TES of genes switching or maintaining coordination pattern between MEF and induced pluripotent stem cells.

Next, we investigated if the degree of allelic coordination is dependent on chromatin accessibility factor enrichment. To explore this, we profiled the enrichment of different chromatin accessibility factors in the gene body and across 2 kb upstream of TSS and 2 kb downstream of TES of highly coordinated, semi-coordinated, and independent genes in MEF and iPSCs (Figs 6 and S4). Interestingly, we found that many accessibility factors such as H3.3, H3K36me3, H3K27ac, H3K4me3, H3K4me2, H3K4me1, and RNAPolII-S2P are highly enriched on highly coordinated genes compared to the semi-coordinated/independent genes in both MEF and iPSC (Figs 6 and S4). Moreover, in iPSC, we found that highly coordinated genes are enriched with chromatin remodeler BRD4 (Fig 6). H3K79me2, H3K9ac, and RNA PolII-S5P were highly enriched to the highly coordinated genes in MEF cells (Figs 6 and S4). Next, we tested if switching of coordination pattern of genes in MEF to iPSC is associated with changes in the pattern of chromatin accessibility factor enrichment. We found that highly coordinated genes in MEF having higher enrichment of H3K36me3, H3K27ac, H3.3, and RNA PolII-S2P compared to the semicordinated genes, which flip their pattern of enrichment in iPSC upon conversion of their coordination pattern (Fig S5A and B). Whereas genes that do not switch their coordination pattern in MEF to iPSC maintain the enrichment pattern of these marks (Fig S5A and B). Altogether, our analysis suggested that higher enrichment of these chromatin accessibility factors ensures highly coordinated allelic bursting. However, other chromatin modifications, such as H3K9me3 and CTCF, did not show such differences in enrichment between different categories of genes (Fig S4).

**Figure S6.**
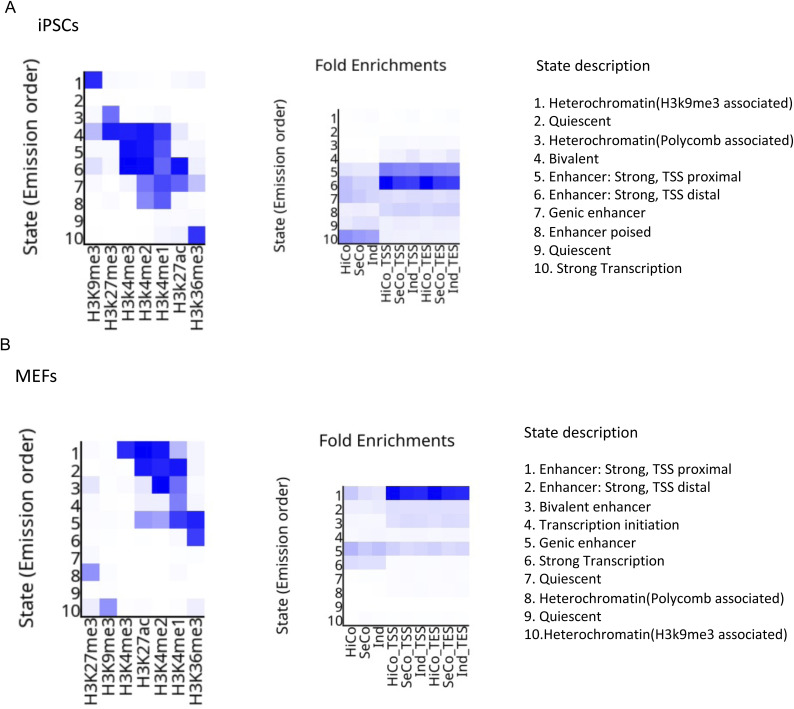
Related to Fig 6. **(A)** Chromatin state profiling through ChromHMM (A) Left: heat map showing the emission parameters in which rows represent 10 different chromatin states based on seven different histone marks (H3K9me3, H3K27me3, H3K4me3, H3K4me2, H3K4me1, H3K27ac, and H3K36me3) in induced pluripotent stem cells. Right: heat map representing the enrichment of different chromatin states in highly coordinated, semi-coordinated, and independent genes and their TSS neighbourhood and TES neighbourhood in induced pluripotent stem cells. **(B)** Left: heat map showing the emission parameters in which rows represent 10 different chromatin states in MEFs. Right: heat map representing the enrichment of different chromatin states in highly coordinated, semi-coordinated, and independent genes and their TSS neighbourhood and TES neighbourhood in MEFs.

Next, we investigated if the chromatin states of highly coordinated, semi-coordinated, and independent genes are different. To explore this, we identified 10 chromatin states in both MEFs and iPSCs using the ChromHMM based on the seven histone marks (H3K27ac, H3K27me3, H3K36me3, H3K4me1, H3K4me2, H3K4me3, and H3K9me3) using the available ChIP-seq datasets (Fig S6). We annotated each state putatively based on related functional element enrichment. Next, we compared the enrichment of these different chromatin states in the gene body in the TSS/TES neighbourhood of highly coordinated, semi-coordinated, and independent genes. Interestingly, we found that chromatin states enriched around strong enhancers showed higher enrichment in highly coordinated genes than the semi-coordinated or independent genes in both MEF and iPSC (Fig S6A and B). Notably, independent genes were highly enriched with bivalent chromatin state compared to the highly coordinated genes in both MEF and iPSC (Fig S6A and B). On the other hand, enrichment of other chromatin states among highly coordinated, semi-coordinated, and independent genes did not exhibit much difference. Taken together, our analysis suggests that enrichment of enhancer elements in highly coordinated genes might play a role in coordinating the allelic transcriptional bursting.

## Discussion

Since the discovery of reprogramming of somatic cells to pluripotent state in 2006, the underlying precise mechanistic aspect of reprogramming remains unknown (Takahashi & Yamanaka, 2006). To fill this gap, quantitatively understanding the transcriptional regulation of reprogramming-related genes is imperative. Emerging trends suggest that transcriptional regulation of two alleles of a gene is not always shared and can be independent, which in turn can lead to temporal expression heterogeneity (Finn et al, 2019; Naik et al, 2021). Therefore, it is important to explore the allelic contribution of genes and their cooperativity to understand how cells fine-tune the optimal expression to bring developmental precision. To understand this aspect quantitatively, we have profiled genome-wide transcriptional burst kinetics at the allelic level and their relevance to cell state transition during iPSC reprogramming. We found that most of the autosomal genes exhibit bursty expression and have dynamic aRME across different stages of iPSC reprogramming (Fig 1), which is consistent with our previous reports in pre-gastrulation embryos (Naik et al, 2021). Importantly, we found that many genes involved in iPSC reprogramming pathways exhibit bursty expression (Fig 1). Interestingly, we found that burst frequency and burst size are highly similar between two alleles for most of the genes across reprogramming (Fig 2A and B). However, in terms of the cooperativity of allelic bursting, we found different patterns of allelic bursting: whereas most of the genes exhibited semi-coordinated allelic bursting, many genes showed highly coordinated allelic bursting (Fig 2C). On the other hand, few genes showed the independent nature of allelic bursting. It is worth mentioning that our analysis of allelic burst kinetics might be erroneous to some extent given the cellular heterogeneity during the iPSC reprogramming. Nevertheless, to reduce this kind of variability, we considered homogeneous populations of cells as clustered through the UMAP analysis. Separately, another limitation of our study is that we have not validated the burst kinetics deduced from the scRNA-seq data through other independent experiments. However, we must mention that many studies have leveraged single-cell RNA-seq technology coupled with allele-specific analysis to profile genome-wide transcriptional burst kinetics (Larsson et al, 2019a, 2019b; Ochiai et al, 2020; Johnsson et al, 2022). Notably, Oichai et al demonstrated that burst kinetics measured through scRNA-seq data can be correlated with kinetics measured through single-molecule FISH (Ochiai et al, 2020). Furthermore, we demonstrate that highly coordinated genes have higher expression level compared to the semi-coordinated and independent genes, suggesting expression level is linked to the degree of coordination of allelic bursting (Fig 3D). In the future, a more precise investigation is necessary to disentangle the interconnection between the coordination of allelic bursting versus expression.

Next, we found that the degree of coordination of allelic transcriptional bursting is relevant to reprogramming pathways. We show that allelic bursting of many genes crucial to iPSC reprogramming occurs in a highly coordinated fashion (Fig 3). We found that genes related to translation, protein stability, protein folding, and RNA processing undergo highly coordinated allelic bursting in iPSC. Specially, ribosome biogenesis-related genes (e.g., *rpl7*, *nop58*, *nmd3*, *nifk*, etc.) become highly coordinated upon initiation of reprogramming (day 8 onwards) and remain highly coordinated through most of the intermediate stages and iPSC (Fig 3). Indeed, reprogramming of ribosome biogenesis is crucial for iPSC reprogramming and stem cell maintenance to enhance translational efficiency (Gabut et al, 2020; Hu, 2020). Moreover, pluripotent embryonic stem cells bear a high density of inactive ribosomes to facilitate increased translation efficiency during their subsequent differentiation into different lineages (Sampath et al, 2008; Novak et al, 2012; You et al, 2015; Gabut et al, 2020). In addition, important genes like *nanog*, *dppa2*, *med28*, etc., which play important roles in iPSC reprogramming, showed highly coordinated allelic expression (Li et al, 2015). Importantly, many genes related to embryonic development and stem cell maintenance showed highly coordinated allelic bursting (Fig 3). Surprisingly, we observed that genes associated with mitochondrial cristae formation (e.g., *ndufa10*) exhibited highly coordinated allelic bursting in iPSC (Fig 3). It is known that iPSC reprogramming is associated with the metabolic shift from oxidative phosphorylation (OXPHOS) to glycolysis, which is accompanied by the formation of immature spherical mitochondria with less dense cristae in iPSCs in comparison to the highly elongated mitochondria with dense cristae network in MEF (Xu et al, 2013; Teslaa & Teitell, 2015; Seo et al, 2018). Although reprogramming to iPSC is associated with the shift from OXPHOS to glycolysis, iPSC remains dependent on mitochondrial metabolism for intermediate metabolites, which play an important role in epigenomic regulation to drive iPSC reprogramming or stem cell pluripotency (Todd et al, 2010; Carey et al, 2014; Moussaieff et al, 2015; Zhang et al, 2016, 2018; Zhou et al, 2016; Guitart et al, 2017; Fang et al, 2019). Therefore, it may be possible that highly coordinated bursting of these mitochondria-related genes is crucial for iPSC reprogramming. Notably, genes involved in nuclear pore formation shifted to highly coordinated state in iPSC. It has been demonstrated that the nuclear pore complex plays an important role in modulating pluripotency and reprogramming (Hansson et al, 2012; Yang et al, 2014). On the other hand, in a recent study, we demonstrated that developmental genes related to gastrulation undergo highly coordinated allelic bursting in pre-gastrulation mouse embryos (Naik et al, 2021). Taken together, we conclude that many genes crucial to reprogramming and development burst in a highly coordinated fashion. Possibly, the coordination of transcriptional bursting between the two alleles fine-tunes gene expression dosage to drive precise development or reprogramming.

Next, we show that epigenomic states contribute to the coordination between allelic transcriptional bursting. We demonstrate that the coordination of allelic bursting is linked to chromatin accessibility. We find that, whereas genes undergoing highly coordinated allelic bursting exhibit equivalent chromatin accessibility between alleles, alleles of semi-coordinated or independent genes have differential chromatin accessibility (Fig 4). Importantly, our analysis suggests that differential chromatin accessibility between alleles of semi-coordinated or independent genes leads to differential binding of certain transcription factors (Fig 5). Previous studies have shown that random chromatin accessibility contributes to transcriptional bursting by providing intermittent accessibility to the transcription factors (Brown et al, 2013; Nicolas et al, 2018; Chen et al, 2019; Fraser et al, 2021). Moreover, open chromatin states are believed to modulate transcriptional burst size and burst frequency (Bartman et al, 2016; Nicolas et al, 2018; Fraser et al, 2021; Bullock et al, 2022). Our analysis extends the support on the role of chromatin accessibility on transcriptional bursting and, most importantly, sheds light on how allelic open chromatin dynamics is linked to allelic transcriptional burst kinetics. In the future, more extensive studies would help to gain deeper insights into the role of chromatin accessibility in mediating allelic bursting coordination.

On the other hand, we find that highly coordinated genes are enriched with important chromatin accessibility factors: H3K4me3, H3K36me3, H3K27ac, H3K4me1, BRD4, and histone variant H3.3 (Fig 6). In fact, H3K36me3, H3K27ac, along with BRD4, have been reported to play a role in orchestrating transcription and burst frequency (Nicolas et al, 2018; Ochiai et al, 2020; Sundarraj et al, 2021; Abe et al, 2022; Altendorfer et al, 2022; Pal et al, 2023). Interestingly, we report the implications of H3.3 in allelic transcriptional bursting for the first time. Moreover, we found that H3K79me2, H3K9ac, RNA PolII-S2P, and RNA PolII-S5P were also highly enriched in highly coordinated genes. Taken together, we propose that these chromatin-related factors play a crucial role in mediating the coordination of allelic bursting. In the future, analysis of allele-specific enrichment of these marks or factors in highly coordinated, semi-coordinated, and independent genes would provide better insight into the plausible regulatory link among allele-specific enrichment of active chromatin marks, burst frequency, and degree of coordination of bursting. Notably, many of these factors or marks have been reported to play an important role in iPSC reprogramming and pluripotency. BRD4 has been shown to play a crucial role in driving the late phase of iPSC reprogramming (Liu et al, 2014).

**Figure 7.**
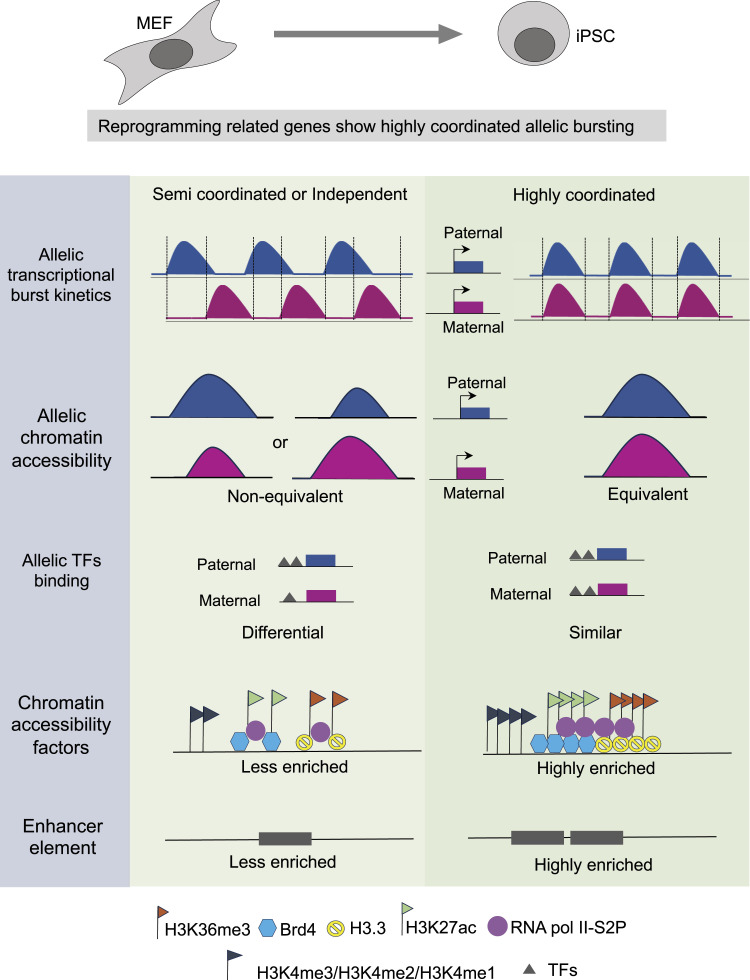
Epigenomic states mediates allelic bursting coordination. Allelic chromatin accessibility is linked to allelic transcriptional bursting coordination. Genes with highly coordinated allelic bursting possess equivalent allelic chromatin accessibility. On the contrary, semi-coordinated or independent genes allelic chromatin accessibility differs. Importantly, highly coordinated genes are highly enriched with chromatin accessibility regulators and enhancer elements.

Occupancy of the H3.3 variant has been attributed to maintaining MEF-specific identity during the early stages of pluripotency reprogramming. However, towards the fag end of reprogramming, they have been conducive to determining and maintaining pluripotent cell fate (Fang et al, 2018). Moreover, H3.3 is found to be critical for early development as its depletion results in early embryonic lethality (Jang et al, 2015). Ablation of H3.3 has been implicated in reducing the overall chromatin accessibility in ESC (Tafessu et al, 2023). Overall, H3.3 enrichment is crucial in pluripotent cell maintenance and early development. Also, the enrichment of active chromatin marks, H3K27ac in enhancer, and H3K36me3 in gene bodies of mouse embryonic stem cells is important for their maintenance (Mikkelsen et al, 2007; Kim et al, 2018). Altogether, we conclude that these chromatin accessibility factors drive reprogramming through mediating allelic bursting coordination of genes involved in iPSC reprogramming to fine-tune the appropriate dosage of these genes (Fig 6). On the other hand, we show that chromatin states linked to enhancer element enrichment are highly enriched in highly coordinated genes (Fig S6). We conclude that enhancer element enrichment in highly coordinated genes might orchestrate the higher coordination of allelic bursting. Indeed, the previous report suggests that enhancer elements can contribute to coordinated transcriptional bursting (Fukaya et al, 2016). Broadly, our study provides fundamental insights into the implications of epigenomic states in fine-tuning of allelic dosage of genes to orchestrate cell fate specification and extends strong support towards the role of epigenomic states in mediating transcriptional bursting (Fig 7).

## Materials and Methods

### Data acquisition

Single-cell RNA-seq, ATAC-seq datasets for MEF to iPSC reprogramming were retrieved from Gene Expression Omnibus under the following accessions: GSE153846 and GSE153844, respectively (Talon et al, 2021). The ChIP-seq datasets used for MEF and iPSC were retrieved from GSE87037 (Aldiri et al, 2017), GSE99592 (Fang et al, 2018), GSE33823 (Yildirim et al, 2011), and GSE90893 (Chronis et al, 2017).

### Allele-specific expression and burst kinetics analysis

To obtain allelic read counts from scRNA-seq data, we performed allele-specific expression analysis following the pipeline as reported previously (Mandal et al, 2020; Naik et al, 2021, 2022). Briefly, we first constructed in silico CAST/EiJ and 129S1-specific parental genome by incorporating CAST/EiJ or 129S1-specific SNPs into the mm10 genome using variant calling file tool (Danecek et al, 2011). Variant calling file was downloaded from the mouse genome project (https://www.sanger.ac.uk/science/data/mouse-genomes-project). Next, we aligned RNA-seq reads into both parental genomes using STAR aligner (STAR-2.7.10a), allowing no multi-mapped reads (--outFilterMultimapNmax 1). We filtered out those genes for allele-specific read counts which had at least two informative SNPs and a minimum of three reads per SNP site. We took an average across SNPs to get gene-level allelic read counts. We normalized the allelic read counts by reads per kilobase million. Because low-expressed genes are dropout prone, we removed low-expressed genes from our analysis to avoid potential dropout effect (Kim et al, 2015; Santoni et al, 2017; Zhao et al, 2017). We considered only those genes, which were expressed in at least ∼27% of cells and had mean expression >10 reads per kilobase million. Allelic ratio was calculated individually for each gene using the formula = (129S1/CAST reads) ÷ (129S1 + CAST reads). A gene was considered monoallelic if at least 95% of the allelic reads came from only one allele. We performed genome-wide allele-specific burst kinetics analysis using SCALE (Jiang et al, 2017). In brief, SCALE relies on Empirical Bayes Framework, which first classifies the genes into monoallelic, biallelic, and silent based on the allele-specific read counts and deduces the allelic burst kinetics based on the two-state model of transcription. It infers different burst kinetic parameters at the allelic level, such as burst frequency (K_on_) and burst size (S/K_off_), as described in the results. We excluded X-linked genes for our burst kinetics analysis. We performed SCALE analysis on each time point of the reprogramming separately.

### Gene ontology

Functional enrichment of different classes of genes was profiled using g:GOSt from gProfiler (https://biit.cs.ut.ee/gprofiler_archive3/e108_eg55_p17/gost) with Benjamini–Hochberg FDR and selected the biological process having FDR < 0.05 from GO: BP (Raudvere et al, 2019).

### Allelic ATAC-seq analysis

For allele-specific ATAC-seq analysis, first we created an “N-masked reference genome mm10” through substituting strain-specific (129S1/SvImJ and CAST/EiJ) SNP position with “N” using SNPsplit_genome_preparation (0.5.0) (Krueger & Andrews, 2016). Strain-specific SNPs were obtained as described above. Next, reads for all samples were mapped to this N-masked genome using Bowtie2 (Langmead & Salzberg, 2012). We removed duplicate reads and mitochondrial reads from our analysis. SNPsplit was then used to create allele-specific BAM files by segregating the aligned reads into two distinct alleles (129S1/SvImJ and CAST/EiJ). Bigwig files were generated from these allelic BAM files using deepTools (version 3.5.1) function bamCoverage (--binSize 100 --smoothLength 500 --normalizeUsing RPGC) (Ramírez et al, 2016). Allelic enrichment metaplots were generated for different gene categories using deepTools functions computeMatrix and plotProfile. Few genes were excluded from our analysis because of the lack of SNP for each day: day 0–15 (of 1,745); day 8–13 (of 1,849); day 9–14 (of 1,906); day 10–19 (of 1,982); day 12A–21 (of 2,521); day 12B–23 (of 2,562); iPSC—14 (of 1,863).

### ChIP-seq analysis

For analysis of chromatin mark enrichment in MEF and iPSC in different categories of genes, we analyzed available ChIP-seq data. For ChIP-seq analysis, the reads were mapped to either mm9 or mm10 genome using Bowtie tool (Langmead & Salzberg, 2012). We removed duplicate reads, mitochondrial reads from our analysis. Different enrichment metaplots were generated for different gene categories using deepTools (version 3.5.1) functions computeMatrix and plotProfile.

### Transcription factor binding analysis using ATAC-seq footprinting

Transcription factor occupancy between individual alleles of genes was predicted using Transcription factor Occupancy prediction By Investigation of ATAC-seq Signal (TOBIAS, version 0.16.0) (Bentsen et al, 2020). Allelic ATAC-seq BAM files were first corrected for insertion bias of the Tn5 transposase using the command ATACorrect for each gene category (highly coordinated, semi-coordinated, and independent) separately. Gene-wise allelic peak regions were identified through allelic peak calling using macs2 (v 2.2.7.1), and peaks were annotated using uropa (v 4.0.3). Next, footprinting scores for individual alleles were generated using the command ScoreBigwig using the ATACorrect data.

Allelic footprint scores were matched to the curated list of JASPAR motifs using the command BINDetect for both alleles separately. Then the resulting scores of each TF motifs for both alleles were correlated using Pearson correlation method.

### Identifications of chromatin states

Chromatin states in both MEFs and iPSCs were profiled using the ChromHMM (v 1.24) based on the seven chromatin marks (H3K27ac, H3K27me3, H3K36me3, H3K4me1, H3K4me2, H3K4me3, and H3K9me3) using the available ChIP-seq datasets (Ernst & Kellis, 2012). In brief, we first constructed binarized bam files of the chromatin marks required as input for ChromHMM using the binarizeBam function of ChromHMM v 1.24. Chromatin state models were generated with the LearnModel function with 10 states. Then we manually curated to assign putative functions to each state according to the combination of epigenomic marks. Finally, enrichment for each category of genes was shown across the states using the OverlapEnrichment function of ChromHMM.

### Quantification and statistical analysis

All statistical analysis was performed using the R software (https://www.R-project.org/). Mann–Whitney two-sided *U* test was used for statistical significance analysis, and *P*-values < 0.05 was considered significant. For correlation analysis, the Pearson test was used.

## Supplementary Material

Reviewer comments
